# Are Medical Students Prepared to Recognise Perinatal Mental Illness? Impact of a Targeted Teaching Intervention on Obstetrics & Gynaecology Placements

**DOI:** 10.1192/bjo.2026.11304

**Published:** 2026-06-30

**Authors:** Anna Lieberman

**Affiliations:** Central and North West London, London, United Kingdom

## Abstract

**Aims:**

To evaluate whether a structured perinatal psychiatry teaching series delivered during Obstetrics & Gynaecology (O&G) placements improves medical students’ awareness of perinatal mental health services, understanding of common and severe perinatal psychiatric conditions, knowledge of psychotropic safety in pregnancy and breastfeeding, and confidence in recognising and managing perinatal mental illness.

**Methods:**

An online perinatal psychiatry teaching series was delivered to 5th-year medical students during their O&G placements. Sessions covered: (1) an introduction to perinatal psychiatry and services, (2) common perinatal psychiatric conditions, (3) severe perinatal mental illness, and (4) psychotropic medication in pregnancy and breastfeeding. Pre- and post-session questionnaires assessed self-reported understanding and confidence using Likert-scale responses. Qualitative feedback explored students’ perceptions of relevance and educational value.

**Results:**

Across the four teaching sessions, 99% of students (85/86) reported the sessions were useful. Following the introductory session, 100% of respondents (34/34) agreed that perinatal mental health teaching should be incorporated into O&G placements, and 85% of students (29/34) reported being at least ‘somewhat likely’ to consider perinatal mental health in their future clinical practice. Significant improvements in self-rated understanding were observed across all sessions. For the introductory session, the proportion of students reporting ‘moderate’ to ‘very good’ understanding increased from 32% (9/28) pre-session to 95% (35/37) post-session. For the session on common perinatal psychiatric conditions, ‘good’ or ‘very good’ understanding increased from 23% (5/22) pre-session to 94% (15/16) post-session. Understanding of severe perinatal mental illness improved from 14% (3/21) of students rating their understanding as ‘good’ or ‘very good’ pre-session to 87% (20/23) post-session. Prior to the psychotropics session, no students reported ‘good’ or ‘very good’ understanding (0/12); following the session, 92% (12/13) rated their understanding as ‘good’ or ‘very good’, with no students reporting ‘no’ or ‘basic’ understanding post-session. Qualitative feedback consistently highlighted the clinical relevance of the teaching and perceived value of the programme.

**Conclusion:**

A structured perinatal psychiatry teaching programme delivered during O&G placements is feasible, highly acceptable to students, and associated with substantial improvements in self-reported understanding and confidence. Embedding perinatal mental health teaching into undergraduate O&G curricula may support earlier recognition of maternal mental illness and strengthen multidisciplinary maternity care.

